# Robust and high resolution hyperpolarized metabolic imaging of the rat heart at 7 t with 3d spectral‐spatial EPI

**DOI:** 10.1002/mrm.25730

**Published:** 2015-05-20

**Authors:** Jack J. Miller, Angus Z. Lau, Irvin Teh, Jürgen E. Schneider, Paul Kinchesh, Sean Smart, Vicky Ball, Nicola R. Sibson, Damian J. Tyler

**Affiliations:** ^1^ Department of PhysicsClarendon LaboratoryUniversity of OxfordEnglandUK; ^2^ Department of PhysiologyAnatomy & GeneticsUniversity of OxfordEnglandUK; ^3^ Department of OncologyCancer Research UK and Medical Research Council Oxford Institute for Radiation OncologyUniversity of OxfordOxfordEnglandUK; ^4^ Division of Cardiovascular MedicineRadcliffe Department of MedicineUniversity of OxfordEnglandUK

**Keywords:** hyperpolarized ^13^C, metabolic imaging, cardiac imaging, cardiac metabolism, pyruvate, pulse sequences

## Abstract

**Purpose:**

Hyperpolarized metabolic imaging has the potential to revolutionize the diagnosis and management of diseases where metabolism is dysregulated, such as heart disease. We investigated the feasibility of imaging rodent myocardial metabolism at high resolution at 7 T.

**Methods:**

We present here a fly‐back spectral‐spatial radiofrequency pulse that sidestepped maximum gradient strength requirements and enabled high resolution metabolic imaging of the rodent myocardium. A 3D echo‐planar imaging readout followed, with centric ordered *z*‐phase encoding. The cardiac gated sequence was used to image metabolism in rodents whose metabolic state had been manipulated by being fasted, fed, or fed and given the pyruvate dehydrogenase kinase inhibitor dichloroacetate.

**Results:**

We imaged hyperpolarized metabolites with a spatial resolution of 2×2×3.8 mm^3^ and a temporal resolution of 1.8 s in the rat heart at 7 T. Significant differences in myocardial pyruvate dehydrogenase flux were observed between the three groups of animals, concomitant with the known biochemistry.

**Conclusion:**

The proposed sequence was able to image in vivo metabolism with excellent spatial resolution in the rat heart. The field of view enabled the simultaneous multi‐organ acquisition of metabolic information from the rat, which is of great utility for preclinical research in cardiovascular disease. Magn Reson Med 000:000–000, 2015. © 2015 The Authors. Magnetic Resonance in Medicine published by Wiley Periodicals, Inc. on behalf of International Society for Magnetic Resonance in Medicine. This is an open access article under the terms of the Creative Commons Attribution License, which permits use, distribution and reproduction in any medium, provided the original work is properly cited. **Magn Reson Med 75:1515–1524, 2016. © 2015 The Authors. Magnetic Resonance in Medicine published by Wiley Periodicals, Inc. on behalf of International Society for Magnetic Resonance.**

## INTRODUCTION

The development of the dissolution dynamic nuclear polarization (DNP) method has allowed hyperpolarized metabolites to probe new frontiers in biomedical research and medical imaging 1. Hyperpolarized [1‐^13^C]pyruvate has been used extensively in vivo to probe cardiac metabolism in basic research 2, 3, 4, 5, and is transitioning to use as a clinical tool that can provide functional information in pathologies where metabolic dysregulation is prevalent, such as heart disease 1 and cancer 6.

While originally dynamic nuclear polarization with [1‐^13^C]pyruvate was used to provide non‐localized spectroscopic information about metabolism at the whole organ level, diseases where metabolic dysregulation is implicated are often spatially localized, such as cancer and myocardial ischaemia. This issue has lead to a variety of strategies to image metabolism following the injection of hyperpolarized [1‐^13^C]pyruvate.

Hyperpolarized cardiac imaging has to overcome at least three key challenges: (A) the heart moves, and this motion is difficult to retrospectively correct for as the observed signal is inherently a function of time, independent of cardiac motion; (B) the paramagnetic nature of blood produces local magnetic field inhomogeneities that make single‐shot imaging techniques more challenging to apply owing to *B*_0_ inhomogeneities, a decrease in $T_{2}^{*}$, and flow artifacts; and (C) any imaging sequence has to be maximally “magnetization efficient,” to avoid destroying the spin structure that dynamic nuclear polarization creates, for example, by an errant $90^{{}^{\circ}}$ pulse. Like any other form of MRI, hyperpolarized imaging is fundamentally a trade off between spectral, spatial, and temporal resolution.

Numerous strategies for hyperpolarized imaging have been proposed. Commonly, two‐dimensional spectroscopic imaging methods are used that either provide a single time‐point image (e.g. with a 12‐s acquisition window and a 12 × 12 matrix 6) or significantly undersample the spectral domain and take an accelerated k‐space trajectory to improve spatial resolution 7. Excitation with two‐dimensional spectral‐spatial radiofrequency pulses may be thought of as representing the limit of spectral undersampling, as only a spectral region defined a priori is excited for subsequent imaging. There has been much interest in using single or multiple‐slice spectral‐spatial excitation pulses for hyperpolarized imaging, and their utility has been shown extensively in the pig heart 8, for saturation‐recovery rate mapping in rat tumor models 9, and in mice 10. The inverse relationship between radiofrequency (RF) pulse length and excitation bandwidth necessitates that most proposed spectral‐spatial pulses are relatively long and, as noted by Sigfridsson et al 11, this puts strenuous requirements on both the maximum strength and slew rate of the gradient set used for imaging. At high field, these requirements effectively preclude the excitation of thin slices. To pursue higher resolution imaging, Sigfridsson et al propose a different multiband approach, in which a temporally short spectral‐spatial pulse excites multiple metabolites with different flip angles and a multiecho readout chain is used for multiple metabolite imaging. The shorter pulse allows for the excitation of a slice whose thickness would not be achievable with conventional pass/stopband single‐excitation pulses. However, as noted by Sigfridsson et al 11, this scheme fails if *B*_0_ is not homogeneous and requires accurate knowledge of the centre frequency of the arriving pyruvate bolus. On account of the oscillatory excitation profile, a small error in this frequency translates into a large error in the flip angle that each metabolite experiences. Pass/stop‐band spectral‐spatial pulses are more tolerant of off‐resonance effects and *B*_0_ inhomogeneity, which improves their utility in vivo.

In this work, we have developed a centric ordered fully three‐dimensional pass/stopband spectral‐spatial echo‐planar imaging (EPI) sequence that enables the imaging of hyperpolarized metabolites with a spatial resolution of $2\times 2\times 3.8{\;\text{mm}}^{3}$, and a (duty‐cycle limited) maximum temporal resolution of 780 ms/3D image. To minimize the effects of gradient infidelity, a fly‐back pulse has been used, whereby RF is only transmitted on the positive lobes of the underlying slice selection gradient. The pulse excites a comparatively large slab (45.5 mm), with subsequent phase encoding in the *z*‐direction trading time for *z*‐resolution. An EPI readout train then follows, with a phase encoding direction that flips on alternate acquisitions to allow for the post hoc correction of frequency shifts and, theoretically, 12 flow and susceptibility artefacts in the resulting cardiac images. The sequence was prospectively cardiac gated and timestamped by a custom hardware device, which prevents an error in the time axis arising from variability in the R–R interval of the anaesthetized rat. This gating reduces the temporal resolution from 780 ms to ∼ 1.8 s/3D image, although the resulting TR is not constant.

To demonstrate the sensitivity of the technique, we have used this sequence to image rodents whose metabolic flux through pyruvate dehydrogenase (PDH) has been modulated by being fed, fasted, and dosed with the pyruvate dehydrogenase kinase (PDK) inhibitor dichloroacetate (DCA). A significant difference in apparent PDH flux was observed in these three circumstances, in accordance with the known biochemistry. We propose that this sequence is a good compromise between spatial and temporal resolutions, and demonstrate that it can effectively image metabolic dynamics in the rodent heart.

## METHODS

### Spectral‐Spatial Pulse Design

**Figure 1:**
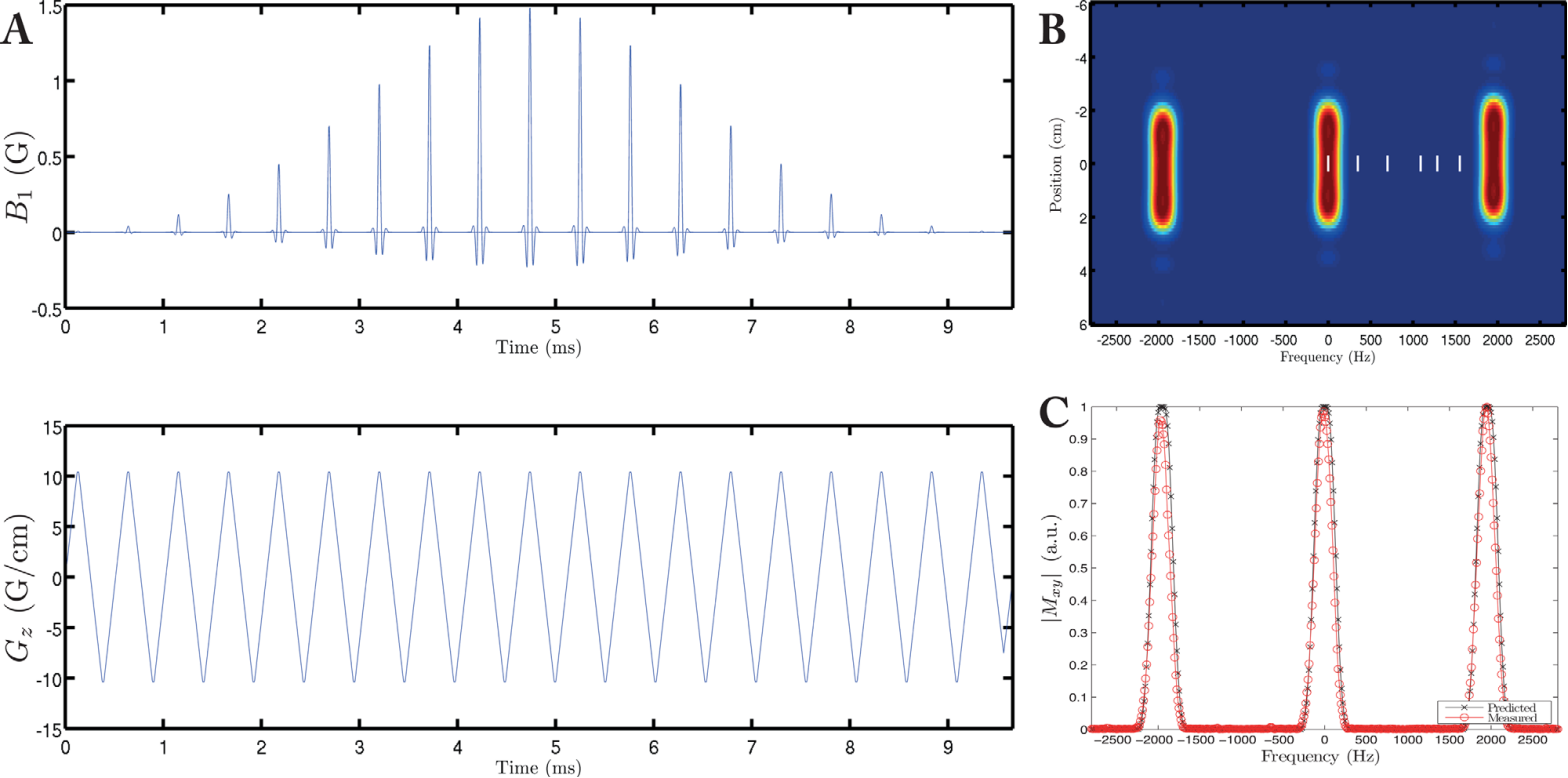
The spectral‐spatial pulse designed for use in this study (**A**) running at $S_{\text{max}}=87.5\;G/\text{cm}/\text{ms}$ has an excitation bandwidth of ∼250 Hz (FWHM), (**B**) and a stop‐band of 2 kHz. The location of the visible resonances following injection of [1‐^13^C]pyruvate are shown as white bars, and are, in order, bicarbonate, urea (a thermal equilibrium sample of which was used as a frequency reference), pyruvate, alanine, pyruvate hydrate, and lactate. The measured frequency profile of the pulse agreed with that predicted (**C**).

As shown in Figure 1a, a 9 ms long pulse was designed under the RF excitation k‐space formalism with zipper correction as described previously 8. Subpulses under a Gaussian envelope were designed with the Shinnar–Le‐Roux algorithm 13, with a time‐bandwidth product of 3 in the spectral domain and 9 in the spatial domain, and zero amplitude on negative gradient lobes to produce a fly‐back pulse. The scheme was slew‐rate ( $S_{\text{max}}$) limited rather than $G_{\text{max}}$ limited, as we found that waveforms played at $S_{\text{max}}$ were reproduced with significantly better fidelity than those at $G_{\text{max}}$, when measured by the method proposed by Duyn et al 14. The pulse had a sublobe duration of 246 μs and 37 sublobes, corresponding to an excitation bandwidth of 240 Hz, and a stop‐band of ∼2 kHz. As shown, this was sufficient at 7 T to avoid every resonance in the spectrum of hyperpolarized [1‐^13^C]pyruvate, denoted as white vertical lines in Figure 1b, and the excitation bandwidth was sufficiently large (3 ppm) to provide relative immunity to transmitting off‐resonance and *B*_0_ inhomogeneity, improving in vivo utility. The minimum excitation slab thickness was obtained when operating at $S_{\text{max}}$, which corresponded to *G_z_* = 10 G/cm and a slab thickness of 45.5 mm. The spectral excitation profile of the pulse was measured on a spherical phantom under the designed conditions; the implementation matched the design (Fig. 1c).

### Pulse Sequence

**Figure 2:**
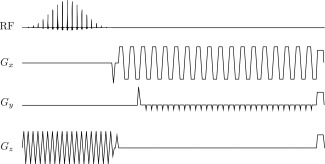
The pulse sequence used in this study, consisting of the spectral‐spatial pulse discussed previously followed by *z*‐ordered phase encoding and a Cartesian *xy* EPI readout with two navigators.

**Figure 3:**
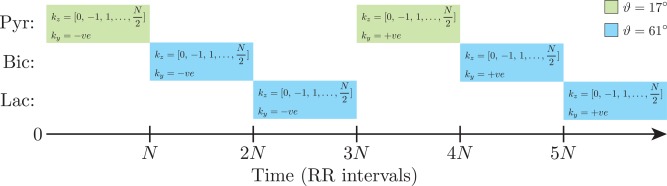
A cartoon of the acquisition strategy. Each imaging block consists of *N* phase encoding steps in the *z* direction, which are ordered centrically. One phase encoding block was obtained per R‐R interval. Three metabolites are acquired in sequential order: pyruvate (Pyr), bicarbonate (Bic), and lactate (Lac). The overall flip angle for each 3D image was ϑ. The in‐plane blipped EPI phase encoding axis, *k_y_*, was alternated on successive acquisitions of the same metabolite.

The above spectral‐spatial pulse excited a 45.5 mm slab of magnetization in the *z*‐direction. Phase encoding with centric‐ordered shots was then used to trade temporal resolution for *z*‐resolution, and an EPI readout with three navigators followed in plane (Fig. 2). The matrix size was 32×32×12 with a field of view (FOV) of $64\times 64\times 45.5{\;\text{mm}}^{3}$, for an acquisition resolution of $2\times 2\times 3.8{\;\text{mm}}^{3}$; TE=16.34 ms; minimum TR=70 ms (duty cycle limited; sequence length =31.48 ms). As illustrated in Figure 3, excitation was performed sequentially across metabolites in the order

$${\;}_{1}^{\;}X_{1}^{1},{\;}_{1}^{\;}X_{2}^{1},\;\ldots ,{\;}_{1}^{\;}X_{N}^{1},{\;}_{2}^{\;}X_{1}^{2},\;\ldots ,{\;}_{2}^{\;}X_{N}^{2},{\;}_{3}^{\;}X_{1}^{3},\;\ldots ,{\;}_{3}^{\;}X_{N}^{3},{\;}_{1}^{\;}X_{1}^{4},\;\ldots$$

where ${}_{\alpha }X_{i}^{j}$ denotes time‐point *j* and phase encoding step i=1…N for metabolite ${}_{\alpha }X$. Here, ${\{}_{1}X,{\;}_{2}X,{\;}_{3}X\}=[\text{Pyr},\;\text{Bic},\;\text{Lac}]$, and *N* = 12. The sequence was electrocardiogram (ECG)‐gated, and the polarity of the in‐plane phase encoding (*y*) axis was alternated on successive acquisitions of the same metabolite. The flip angle per shot was chosen to be $\theta =3^{{}^{\circ}}$ for X=Pyr, and θ=20° for X≠Pyr, such that the overall flip angle for each 3D image is $\vartheta =\arccos(\cos^{N}\theta )=17^{{}^{\circ}}$ for X=Pyr and 61° for X≠Pyr.

A thermally polarized 5 M ^13^C urea phantom was included next to the animal to provide a carbon frequency reference. Three‐dimensional, cardiac‐gated volume shimming was performed using the previously mapped behavior of the magnet's shim set and a spherical harmonic cardiac autoshimming algorithm as described previously 15. Multiecho cardiac gated gradient echo *B*_0_ maps were acquired (48 × 48 matrix; 70 × 70 mm FOV; 1.2 mm slice; TR=100 ms; TE=3.06, 3.56 ms; *T* ${}_{\text{read}}=1$ ms; total scan time 60 s) to verify homogeneity over the whole volume of acquisition 15. The ^13^C centre frequency $\omega_{\text{transmit}}$ was set by identifying the frequency of the urea phantom $\omega_{\text{urea}}$, and compensating for any small measured *B*_0_ variation between the urea phantom and the heart $\Delta B_{0}$ as $\omega_{\text{transmit}}=\omega_{\text{urea}}+\Delta B_{0}\gamma_{{}^{13}C}/\gamma_{{}^{1}H}$.

Anatomical images were acquired with a gradient echo cine sequence described previously 16, with the same slice thickness and FOV as the hyperpolarized data ( TR=4.6, TE=1.41 ms, 28 frames, FA =25°).

### Hardware

Experiments were performed on a Varian 7 T DDR system with *G*_max_ = 17.5 G/cm, *S*_max_ = 87.5 G/cm/ms, with an actively detuned transmit/surface receive setup consisting of a 72 mm dual‐tuned ^1^H/^13^C proton/carbon birdcage volume coil with a 40 mm two‐channel ^13^C surface receive array with an integrated preamp (Rapid Biomedical GMBH, Rimpar, Germany). A home‐made combined RF shield and animal handling system was required to ameliorate undesired electrostatic standing wave interactions occurring inside the magnet bore. The volume coil ^13^C flip‐angle calibration was performed on a cylindrical reference phantom consisting of 500 mL of 1 M natural abundance urea with thermal equilibrium slice‐selective spectroscopy. It was found that the position and width of the resonant frequency of the volume coil did not change appreciably between loading with the phantom and with a rat, indicating that the delivered carbon flip angle was as calibrated. The $B_{1}^{+}$ homogeneity of the volume coil was assessed by acquiring a multiple flip angle *B*_1_ map on the urea phantom with TR at least 5*T*_1_. We found that the spatial variation of flip angle was 8%, which is believed to be dominated by noise in the acquired data.

To allow for the reconstruction of an accurate time axis, it was necessary to create a simple hardware device that recorded when each ECG‐gated scan occurred to a resolution of 10 μs. This high temporal resolution ensured that the error on recording the time at which each shot occurs could be neglected. Scans were gated to the R‐wave using a threshold‐based detection device described previously 16. Respiratory gating was not performed, as no artefacts were observed that were believed to be due to respiratory motion. The period of respiratory motion (c. ∼ 1.5–2 s in the anaesthetized rat) was comparable to the duration of each acquisition.

### Animal Handling and Dosing

All experiments were performed in accordance with relevant UK legislation (with personal, project and institutional licenses granted under the Animals (Scientific Procedures) Act 1986), and were subject to local ethical review and an independent cost‐benefit analysis.

Approximately 40 mg of [1‐^13^C]pyruvate doped with 15 mM OX063 trityl radical and 3 μL of a 1 in 50 dilution of Dotarem (Guerbet Laboratories Ltd) gadolinium chelate was polarized and dissoluted in a prototype polarizer as described previously 17. The estimated liquid state polarization was ∼32% after dissolution, 2 mL of the resulting 80 mM pyruvate solution were injected manually over a 20 s period through a previously placed tail vein cannula in anaesthetized male Wistar rats (2% isoflurane in $90\%{\;O}_{2}$, 10% N_2_O; *n* = 4; body weight 410 ± 70 g). Pyruvate was injected approximately 10 s after dissolution, and its pH was measured after each dissolution to ensure correct neutralization in all experiments. Animals were housed in a custom handling system within the magnet bore that regulated their temperature and provided RF shielded electrical connections for ECG triggering and the RF coils. The scan was started prior to the dissolution of hyperpolarized pyruvate.

Four rats were scanned on three separate occasions with at least two days as a recovery period between scans. Animals were imaged three times: (A) in the fed state after being allowed food and water ad libitum; (B) after being fasted for at least 8 h prior to imaging; and (C) following injection with the PDK inhibitor, DCA, in the fed state. Rats were dosed with DCA at approximately 30 mg/kg as previously proposed 18, 30 min before hyperpolarized imaging. To minimize the total volume of injected agents, DCA was administered as a 100 μL bolus of neutralized 10% DCA solution at pH 7.2–7.8.

### Reconstruction

Data were regridded appropriately in MATLAB, and linear phase correction performed in‐plane on the EPI readout. Alternate lines of k‐space were then Fourier transformed independently, and added in magnitude in image space; halving the FOV and eliminating the ghost. The noise decorrelation matrix for the two‐channel array was calculated following the method of Kellmen and McVeigh 19, and the data prewhitened. Images were combined and coil sensitivities calculated following the method of McKenzie 20. Following the method of Cunningham et al 21, blip‐reversed EPI data were spatially corrected by a mutual information maximization method, to compensate for small spatial shifts caused by being off‐resonance. Data were prefiltered with a Chebyshev kernel before computation of the mutual information metric.

The resulting five‐dimensional image stacks were zero‐filled by a factor of two in all spatial directions, producing an image stack with a final in‐plane resolution of $1\times 1\times 1.9{\;\text{mm}}^{3}$. Data were then overlaid on anatomic images for reference. A manually segmented ROI was drawn in the lumen (pyruvate) or myocardium (bicarbonate, lactate) and the ratio of maximum bicarbonate to maximum pyruvate computed after subtracting the mean noise as measured from the last 10 time points of the experiment.

## RESULTS

**Figure 4:**
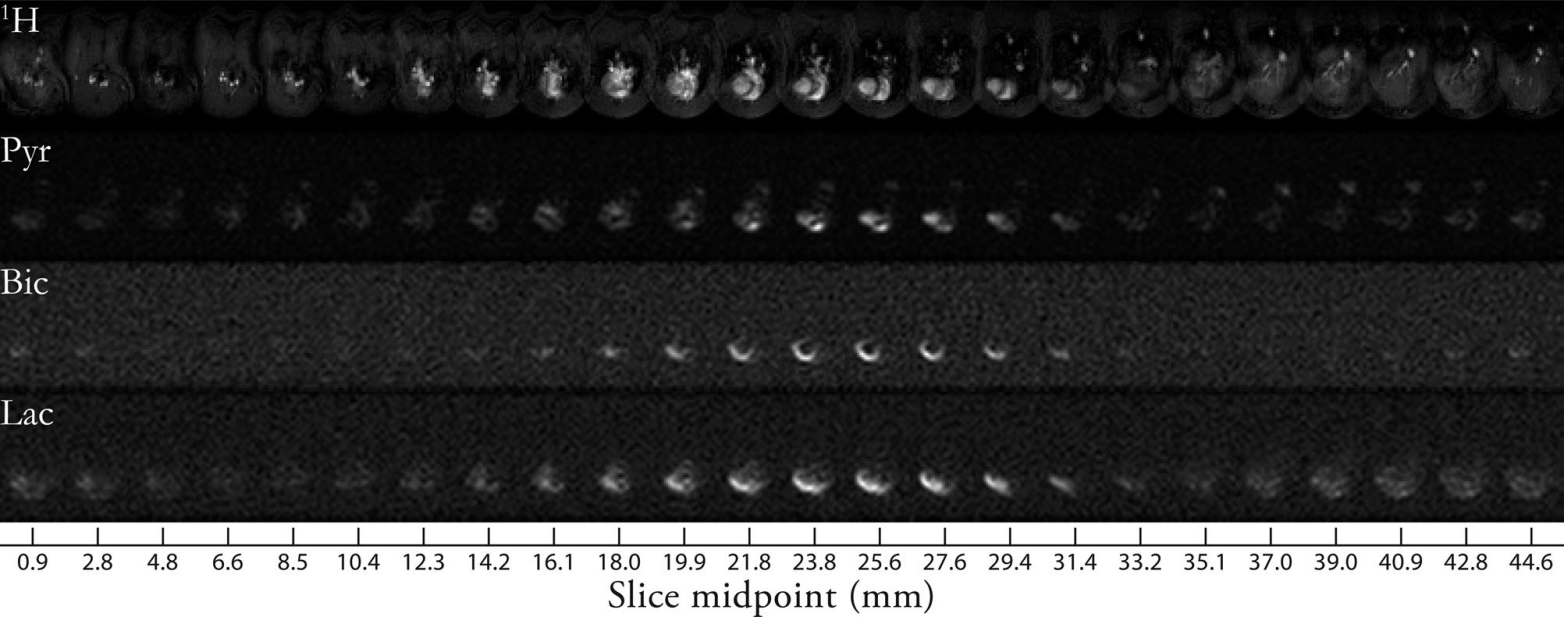
Representative axial maximum intensity projections of data acquired from animals fed and dosed with DCA. Images shown concatenated with *z* decreasing from left to right; the reference cine images (top) span the neck (top left), heart (top middle), and anterior lobes of the liver (top right). Pyruvate was resolved in the ventricles of the heart, and additionally in the inferior vena cava. The production of hyperpolarized bicarbonate occurred predominantly in the myocardium, and in this instance it was not possible to resolve metabolism in segments distal from the chest wall. Hyperpolarized [1‐^13^C] lactate was also detected after production in the myocardium, and, to a far lesser extent, the liver.

We were able to resolve the metabolism of prepolarized [1‐^13^C]pyruvate in the in vivo rat heart with a reconstructed spatiotemporal resolution of $1\times 1\times 1.9{\;\text{mm}}^{3}$ and ∼1.8 s. The three‐dimensional sequence reports on hyperpolarized metabolism in a $64\times 64\times 45.5{\;\text{mm}}^{3}$ FOV, which was sufficient to encompass the whole heart and the liver. The large field of view was advantageous as we obtained information from multiple organs at the same point in time. The data were five‐dimensional and an example maximum intensity projection (summed over time) showing the acquisition volume is illustrated in Figure 4.

**Figure 5:**
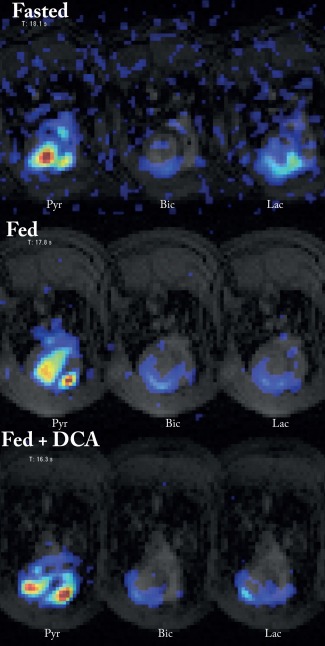
Representative midventricular single‐slice single time‐point data overlaid on anatomical reference images. Shown here are the three metabolites (pyruvate, bicarbonate, and lactate) at the timepoint of maximum pyruvate signal, in the three groups of rats. The color axis for each set of three images is scaled relative to the maximum pyruvate. These data are stills from Supporting Information Videos S1–3.

Alternating the EPI blip direction allowed for the accurate correction of bulk frequency shifts, and the data overlay with anatomical cine images, illustrating myocardial metabolism (cf. Fig. 5). The time‐courses of these data are available as Supplementary Videos S1–3, which are representative examples from each group of rats scanned. The *B*_1_ homogeneity arising from the volume transmit/surface receive hardware used was sufficient to ensure a uniform excitation across the volume of interest. This homogeneity should be contrasted to the spatially nonuniform flip angle arising from a surface transmit/receive coil. However, due to the sensitivity profile of the surface receive coil used, it was not always possible to resolve segments of the myocardium distal from the chest wall. Across the 12 scans, the distal wall was completely resolved twice, partially resolved four times, and not resolved six times.

**Figure 6:**
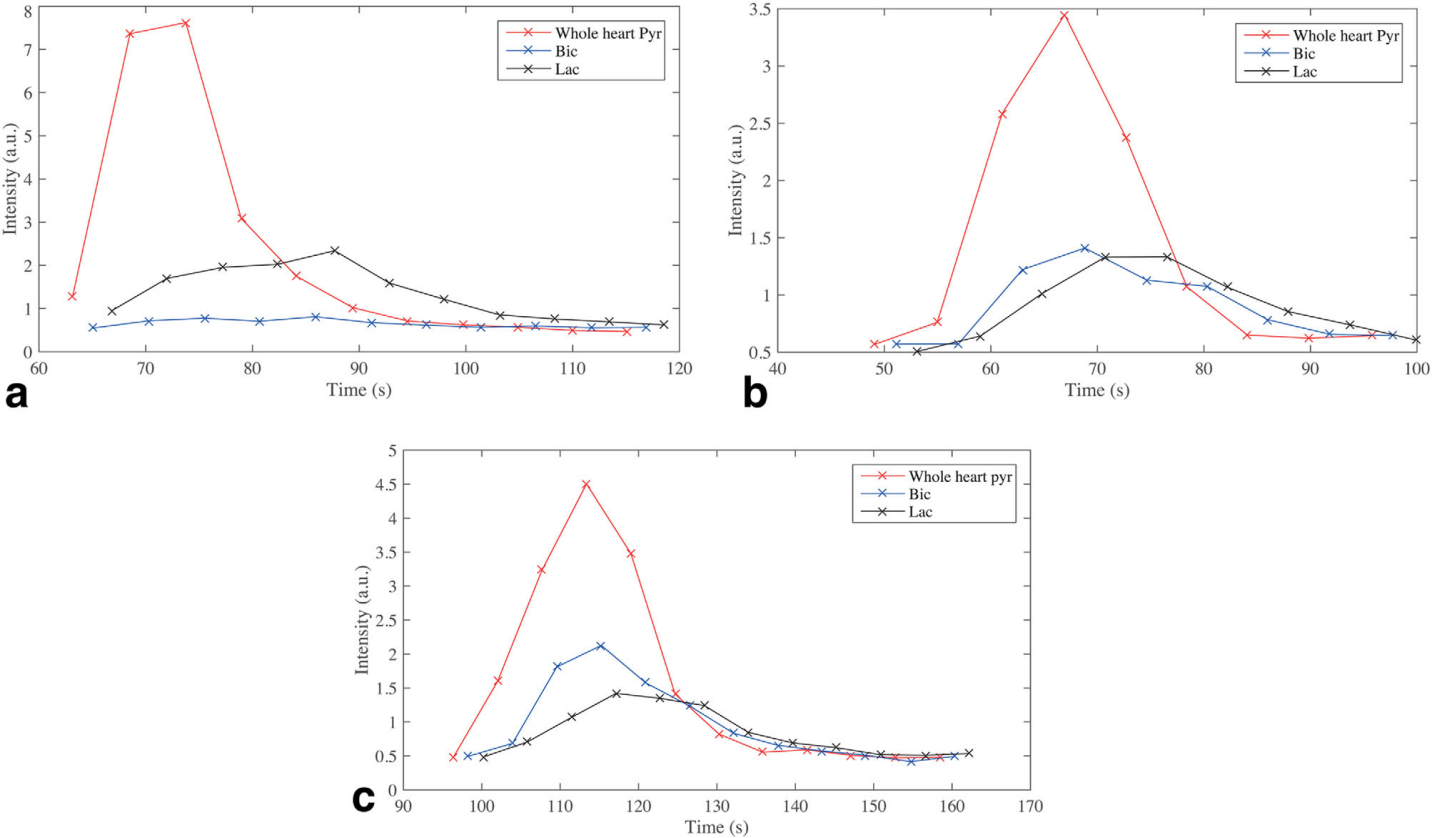
Representative single‐slice whole‐heart time courses acquired from fasted animals (**a**), fed animals (**b**), and animals fed and administered DCA (**c**).

The temporal resolution of the technique was sufficient to resolve metabolic dynamics following injection of hyperpolarized [1‐^13^C]pyruvate in a single voxel. Concomitantly with the known anatomy, pyruvate was observed perfusing the great vessels at early times, before arriving in the right and then left ventricles. Myocardial bicarbonate and lactate production subsequently followed. Summing the data acquired in a manually segmented ROI over the heart allowed for the reconstruction of metabolic dynamic time‐courses (Fig. 6), as expected.

**Figure 7:**
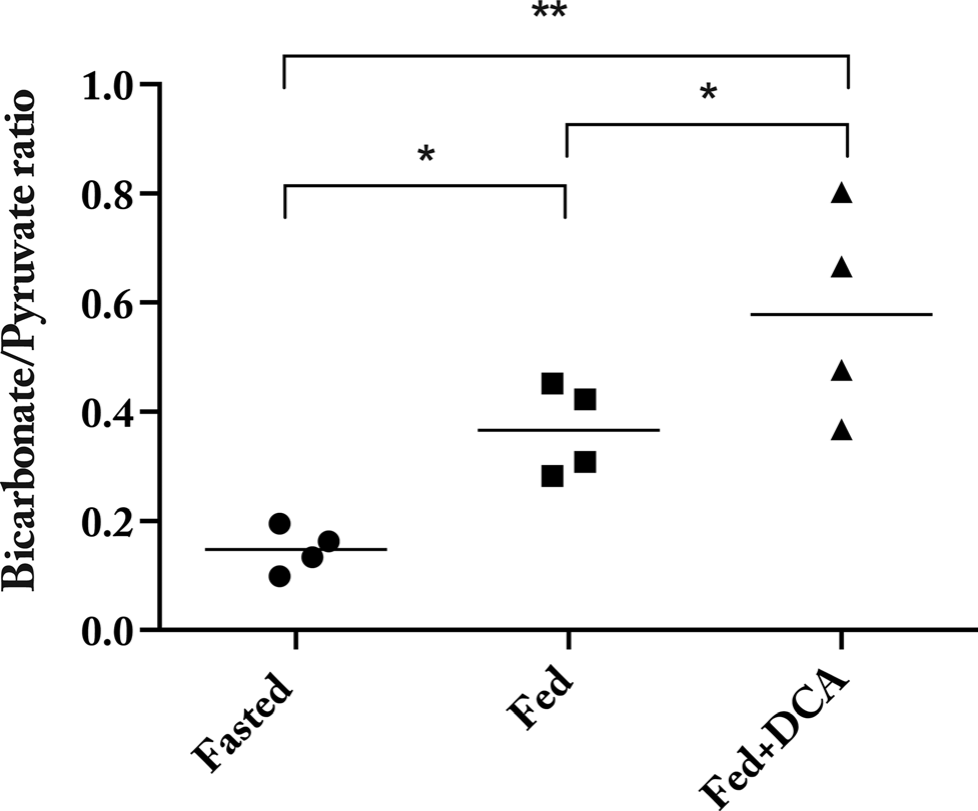
The ratio of maximum bicarbonate/pyruvate was significantly different across the three groups of animals scanned, reflecting the known inhibition of PDK by DCA. ( * denotes *P* < 0.05; $**\;P<10^{-5}$.)

Reflecting the known modulation of PDK by both DCA and fasting, significant differences in the ratio of the summed myocardial maximum bicarbonate to maximum pyruvate were observed across the three groups of animals scanned (Fig. 7; Student's *t*‐test *P* = 0.03 between fasted and fed, and fed and DCA; *P* = 0.0008 between fasted and DCA). The administration of DCA serves to approximately double this ratio compared to the fed animals, and quadruple it compared to fasted animals (DCA to fed: 1.6 ± 0.3, DCA to fasted: 3.9 ± 2.8; values ratio means ± S.E.M. propagated in quadrature). This doubling of PDH flux between fed animals and those dosed with DCA is the same as that obtained when this experiment was performed by simple unlocalized ^13^C spectroscopy following hyperpolarized [1‐^13^C]pyruvate injection 18.

**Figure 8:**
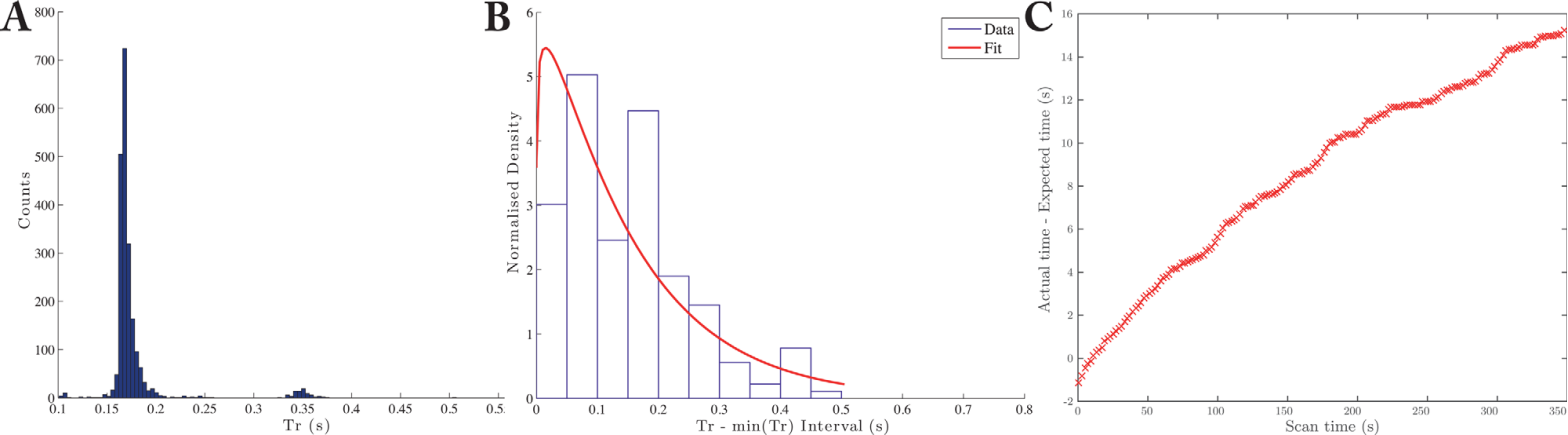
a: The distributions of gated shot TR was distributed around the R–R interval (here ≈170 ms), with a small number of missed triggers (at ≈350 ms). **b**: As expected for a stochastic waiting process, the subsequent distribution of image TR was described by a gamma distribution (fit), with a large spread in values (min/max TR/image =1.966 s /  2.418 s). **c**: The error between the expected ( N× R–R interval) and measured scan times becomes progressively worse as time increases.

A slight distribution of acquisition TRs was observed (Fig. 8a), with a large peak at the R–R interval and a small number of shots at 2× R–R interval, reflecting a number of missed triggers that we conjecture are due to respiratory motion. It was noted that the TR of the experiment was not constant, and that the distribution of 3D image TRs was well described by a gamma distribution (Fig. 8). The mean of this distribution was not equal to the number of *z* phase encodes, *N* × the R–R interval. The observed coefficient of variation of TR/image was 40.4±11.4% (distribution σ/μ ± S.E.M. over 16 scans), reflecting a significant variability in the R–R interval of the anaesthetized rat. We are confident that this effect is biological in origin and not an experimental artefact; the variability vanished when a signal generator was used to provide an input comparable to an ECG into our cardiac triggering hardware. We note that the correct time axis is fundamentally important in hyperpolarized imaging, as it is desirable to create quantitative maps of metabolic rate constants of interest.

## DISCUSSION

We have developed a novel sequence for the spectroscopic imaging of hyperpolarized [1‐^13^C]pyruvate and its downstream metabolites in the in vivo rat heart. The proposed sequence was robust in the sense that (A) the fly‐back 3D spectral‐spatial excitation reduces gradient demands, and was replayed with better fidelity than pulses that excite a thin slab, (B) the excitation bandwidth of the pulse was relatively large (3 ppm), minimising sensitivity to *B*_0_ inhomogeneity, and (C) bulk frequency shifts on the order of 30 Hz that arose from uncertainty of the pyruvate frequency a priori could be corrected for with the alternating EPI readout.

Our hardware produced accurate information about the acquisition time of each image, which was used to compensate for the variability of the heartbeat of the anaesthetized rat. Cardiac small animal imaging is challenging, as the required resolution and rapid heart rate increases both gradient hardware and SNR demands. Nevertheless, it is desirable to image small rodents as they form the predominant animal model of choice for cardiovascular research, and unlike large animals, mice (and increasingly rats) can be readily genetically manipulated to further probe the genetic basis of disease 22. Therefore, while small animal imaging is challenging, it is of considerable utility owing to the large number of different conditions that could be investigated. It is also a central tenant of ethical animal experimentation to minimize the degree of discomfort experienced using a less sentient model species if one is available 23.

Additionally, small animal hyperpolarized cardiac MR can be considered a “worst‐case” scenario for human imaging, as the required spatial and temporal resolutions are in excess of those required for human use. As the total magnetization available for hyperpolarized imaging is independent of the scanner hardware (instead depending on the polarizer used and the quantity of visible carbon injected), the ultimate SNR limited resolution for a given sequence and a given quantity of visible carbon will also be largely independent of subject and hardware. For the reasons outlined above, it is therefore desirable to develop the best possible imaging sequence for capturing hyperpolarized metabolism in the rodent heart, even though scaling arguments predict that this is more challenging than imaging larger animals, including humans.

The proposed sequence is a good compromise between spectral, spatial, and temporal resolution. As shown, we were able to spatially resolve metabolism within the myocardium and liver with temporal resolution sufficient to capture metabolic dynamics of interest. The fly‐back spectral‐spatial excitation effectively represents the limit of spectral undersampling and reduces demands on gradient hardware. We could record metabolism in multiple organs simultaneously, with an acquisition resolution of $2\times 2\times 3.8{\;\text{mm}}^{3}$ in a comparatively large FOV that ensures whole heart coverage. Moreover, the large excitation slab reduced flow artefacts that can occur due to pyruvate in the blood flowing into (and out of) a thin excitation slab for 2D multislice imaging approaches. We do not observe any in‐plane flow ghosting artefacts, reflecting the comparatively short readout train as EPI readouts are known to be particularly prone to these distortions 24. The resolution of this sequence compares favorably to other approaches, such as multiband, 11 IDEAL CSI 25, high resolution accelerated spiral CSI 26, spectral‐spatial excitation followed by a multislice spiral readout 8, or *k*‐*t* undersampling following spectral‐spatial excitation with an EPI readout 27, for which the reported resolutions are $2\times 2\;\times 4{\;\text{mm}}^{3},\;4.4\times 4.4\times 10{\;\text{mm}}^{3},\;2.7\times 2.7\times 10{\;\text{mm}}^{3}$, $8.8\;\times 8.8\times 10{\;\text{mm}}^{3}$, and 1×1×8 mm^3^, respectively. While these sequences have been developed on a variety of hardware with different limitations, and utilized on a variety of different animal models of disease, they all fundamentally encounter the SNR limitations of pyruvate *M*_0_ generated in a 3 T preclinical hyperpolarizer. An argument can, therefore, be made that the ultimate achievable resolution of hyperpolarized pyruvate imaging depends on the quantity of visible carbon injected, which is approximately constant, and the compromises made in the design of sequences to image it. We accordingly believe that the proposed sequence represents an acceptable compromise between spatial, spectral and temporal resolution that will be generally applicable to those wishing to image hyperpolarized metabolism in any organism. The scanner used is a common preclinical system, and is not state‐of‐the‐art. Accordingly, the gradient waveform under the spectral‐spatial pulse is triangular. We have found that the fly‐back pulse running at $S_{\text{max}}$ produces a waveform whose biggest distortion is a constant time offset, which can be corrected by the introduction of a small delay between the RF relative to the gradient waveform, and whose effect otherwise is to slightly degrade the slice profile. This should be contrasted to non‐fly‐back spectral‐spatial pulses, where infidelities cause spurious excitation at 0 Hz, producing unwanted mixed metabolic excitation. The duration of the fly‐back pulse is chosen to have a narrow spectral passband and a large stopband, that is, to avoid exciting pyruvate hydrate and lactate at the same time. The pulse proposed here is accordingly more tolerant of gradient fidelity issues. Changing the performance of the gradient set serves to adjust the excitation slab thickness. Reducing the slab thickness increases resolution at the cost of gradient requirements and SNR; increasing it reduces either the spatial or temporal resolution with associated SNR and gradient requirement benefits.

As expected, the sensitivity to metabolism of this hyperpolarized imaging strategy was confirmed by modulating the metabolic state of the rodents imaged, whereupon significant differences in PDH flux were observed between animals that were fasted, fed, and fed and given the PDK inhibitor DCA. The fact that the relative increase in apparent PDH flux was the same as when the same experiment was performed spectroscopically validated the technique.

Three dimensional hyperpolarized imaging strategies need to be designed to take into account the effect of the depletion of the longitudinal magnetization by each phase encode on the resulting image. The relative SNR efficiency of 3D pulses for hyperpolarized imaging is well documented 28, 29, and can be approximated by the ratio

(1) $$\frac{{\text{SNR}}_{2D}}{{\text{SNR}}_{3D}}=\frac{\sin\vartheta_{2D}}{\sqrt{N}\;\;\sin\theta_{3D}}$$

where *N* is the number of *z* phase encodes. Here *N* = 12, $\vartheta_{2D}=17{}^{\circ}$ or 61°, and $\theta_{3D}=3{}^{\circ}$ or 20°. This predicts an approximately equal SNR efficiency for pyruvate imaging (2D/3D ratio = 0.993), and a ∼25% SNR benefit for metabolites other than pyruvate (ratio = 0.743). This SNR improvement from exciting a large volume comes at a cost, as noted by Wild et al, as the effect of the depletion of the longitudinal magnetization is to apply a filter in *k_z_* that serves to blur the image through the *z* direction. The size of this effect is related to $1-{\left(\cos\;\theta \right)}^{N}$, the acquisition scheme, and the ratio $T_{r}/T_{1}$ that together determine the Full Width Half Max (FWHM) of the filter. The quantity $1-{\left(\cos\theta \right)}^{N}$ here is small ( ∼0.02) for pyruvate and ≈1/2 for metabolites other than pyruvate. To quantify the effect of this proposed acquisition scheme, the *k_z_* filter *H*(*z*) as defined by Wild et al was numerically calculated for the 3D sequence described here (i.e. with *N* = 12 centric ordered shots), and Fourier transformed to find the point spread function. The resulting *k_z_* filter had a FWHM of 1.012 for pyruvate, and 1.176 for metabolites other than pyruvate. We consider a subvoxel blurring in the slice direction to be outweighed by the ∼25% SNR gain resulting from the three‐dimensional approach.

The error in gated scan timing, here defined as the difference between actual and “expected” (*N* × R‐R interval) scan time, became progressively worse over time (Fig. 8c). The mean of this distribution was not equal to *N* × R–R interval; and it was broad: the coefficient of variation of TR/image was 40.4±11.4% (distribution σ/μ± S.E.M. over 16 scans). As shown in Figure 8, the observed distribution of TR s were well‐described by the two‐parameter Gamma distribution

(2) $$f(x;k,\theta )=\frac{x^{k-1}e^{-\frac{x}{\theta }}}{\theta^{k}\Gamma (k)}\;\;\text{for}\;x>0\;\text{and}\;k,\theta >0$$

where Γ(k) is the gamma function $\Gamma (k)=\int_{0}^{\infty }x^{k-1}e^{-x}\;dx$, and *k* and *θ* are the shape and scale parameters that were fit to the acquired data. As the ECG can be considered a stochastic time‐series that is a sum of underlying fundamental processes 30, 31, the scan counts are positive semidefinite, and the distribution of increment times, that is, TRs, are Gamma distributed, the number of gated scans at a given time can be considered a Gamma process, a type of generalized Poisson process 32. This fact implies that dynamic techniques which neglect this information will have a skewed time axis that becomes progressively distorted as time increases, despite the relatively small distribution in the R‐R interval itself. Computed “system independent” quantities of interest such as rate constants or perfusion values, which fundamentally depend on an accurate time axis, are therefore liable to be biased if data is not time‐stamped accurately. We note that the majority of preclinical scanners do not record the exact time at which a shot occurs. Future work might be able to exploit the distribution of repetition times in this acquisition with sequences inspired by stochastic NMR 33.

Owing to the fact that *z*‐phase encoding is centric ordered, we propose that the effective timepoint for each 3D volume acquired is defined as the echo time of the first shot, when the centre slab of *z* k‐space is acquired. We note that this definition might be contentious, but the use of an accurate timestamping device allows for the post hoc reconstruction of the time axis as required; here, the axis was defined as above. The acquisition strategy spans several orders of magnitude in time, with the pulse length (= 31.48 ms) short in comparison to the cardiac cycle (∼ 150 ms) and respiratory motion ( ∼1.5−2 s). We did not observe any artefacts that could be ascribed to respiratory motion, but a separate acquisition showed that cardiac triggering was still required to avoid motion artefacts in the myocardium.

### Limitations

There are several potential limitations to this 3D approach for hyperpolarized imaging. First, the SNR benefit and the width of the *k_z_* filter arising from the 3D approach is highly dependent on the actual flip angle delivered, and therefore requires good $B_{1}^{+}$ homogeneity. In this study, a volume transmit coil was used that was homogeneous over the region of interest. It is likely that this acquisition strategy will be challenging with a surface transmit/receive coil, where the flip angle delivered is inherently a function of space. We would expect the resulting images to have a significant degree of interslice blurring arising from the large flip angle close to the surface of the coil. However, if these effects can be ameliorated by ensuring homogeneous $B_{1}^{+}$ (as they have been here), the 3D approach can fundamentally deliver better SNR.

Second, hyperpolarized imaging sequences that use phase encoding effectively apply a blurring filter in the phase encoding direction, here *z*, due to the depletion by each shot of the initial magnetization. This effect is proportional to $1-\cos{\left(\theta \right)}^{N}$, which grows quickly with *θ* and *N*. However, the SNR benefit grows only as $\sqrt{N}\;\;\sin\theta$, which is approximately linearly at small *θ*, and slowly with *N*. There are, therefore, several regions of *θ* and *N* where this approach is inferior to 2D imaging with multiple slices, for example, as $\theta \to 90^{{}^{\circ}}$, where the slice resolution approaches the FOV, and at very low flip angles and small *N*, where the 2D approach has better SNR. As demonstrated, we have chosen values of *θ* and *N* such that this effect was ameliorated, and a substantial SNR benefit was obtained at the cost of a modest trade‐off in slice resolution.

Third, one would expect that the high slew‐rate requirements of the fly‐back pulse would introduce nonlinear distortions that serve to degrade the excitation profile of the pulse. However, this has not proved to be the case (c.f. Fig. 1c), despite the prediction of a slight problem with pre‐emphasis from the measured gradient impulse response function for this pulse.

Fourth, the 3D sequence is relatively robust to flow artefacts, as the large excitation slab thickness ensures that excited spins will be read out irrespective of their motion. However, we have not quantified the effect of flow over the 12 RR intervals that each volume is acquired over, and it is likely that different parts of *k_z_* correspond to flowing spins in different positions, which could result in a flow artefact. We expect two types of artefact to arise from flow; ghosting in the phase encoding direction arising from in‐plane motion during the EPI readout 24, and linear shift in *z* arising from through‐plane motion during the 3D readout. This last effect arises as the *k_z_* gradient blips can serve to act as velocity encoding gradients, creating a phase ramp on spins through *z*. In the proposed sequence, the largest blip is 328 μs long, and 7.51 G in amplitude. This corresponds to a large velocity encoding moment; spins flowing at 23 m/s would be shifted by one voxel in *z*. This artefact is correspondingly negligible in the rat heart.

It is also appropriate to consider the effect of metabolism on the resulting images, as the ^13^C label is transferred to downstream metabolites during the acquisition period of the 3D volume. This effect is analogous to modulating the *T*_1_ of the compound imaged, as the net effect of the (dis)appearance of a small quantity of hyperpolarized metabolite in bulk will be analogous to the increase or decrease in *T*_1_ for that compound. Accordingly, the effect on the image is a slight blurring or sharpening through *k_z_*, as the nonrenewable magnetization is depleted or slightly replenished between phase encodes. We do not observe any appreciable through‐plane blurring, indicating that this effect is small.

Finally, the first‐order phase correction method used did not always remove the Nyquist ghost arising from the EPI readout train used. Owing to the large acquisition FOV of the technique, we elected to recombine odd and even lines separately in every case, halving the FOV and eliminating the ghost. However, in general it is possible to perform a 2D phase correction to remove the distortion 34. Unfortunately, the dynamic nature of hyperpolarized imaging means that many standard algorithms will not work “out‐of‐the box” and instead a manual in‐plane phase map has to be created and applied for each acquisition, estimated from high SNR images that cover as large a volume as possible, including both ventricles and the myocardium. This is a potentially challenging and time‐consuming step that might drive a desire to perform fly‐back EPI.

## CONCLUSIONS

We have developed a novel ECG‐gated three‐dimensional pulse sequence for the cardiac spectroscopic imaging of hyperpolarized [1‐^13^C]pyruvate and its downstream metabolites in vivo. The sensitivity of the developed sequence to metabolism has been confirmed by manipulating the metabolic state of the rodents imaged. The 3D spectral‐spatial excitation is relatively immune to *B*_0_ inhomogeneity, and does not place unphysical demands on gradient hardware to image at high spatiotemporal resolution.

A simple argument is presented to support the hypothesis that three‐dimensional hyperpolarized imaging is more SNR efficient than multislice approaches, at the cost of a slight blurring in the slice direction. We have further shown that cardiac time‐stamping is important when imaging dynamic processes because of the stochastic nature of cardiac gating, a fact that is often overlooked. This work allows the spectrally efficient metabolic imaging of cardiac metabolism in small animals at high spatiotemporal resolution. This is of utility for preclinical research into both cardiovascular disease and more broadly diseases where metabolism is dysregulated.

## Supporting information


**Video S1:** Representative mid‐ventricular single‐slice time‐resolved data overlaid on anatomical reference images. Shown here are the three metabolites, from left to right, pyruvate, bicarbonate and lactate in a fasted rat. The color axis for each of the three images is scaled relative to the maximum pyruvate.

**Video S2:** Representative mid‐ventricular single‐slice time‐resolved data overlaid on anatomical reference images. Shown here are the three metabolites, from left to right, pyruvate, bicarbonate and lactate in a fed rat. The color axis for each of the three images is scaled relative to the maximum pyruvate.

**Video S3:** Representative mid‐ventricular single‐slice time‐resolved data overlaid on anatomical reference images. Shown here are the three metabolites, from left to right, pyruvate, bicarbonate and lactate in a fed rat administered the PDK inhibitor DCA. The color axis for each of the three images is scaled relative to the maximum pyruvate.

Supporting Information
