# Supplementary material for: Robust and high resolution hyperpolarized metabolic imaging of the rat heart at 7 t with 3d spectral‐spatial EPI
Source: Magn Reson Med. 2015 May 20;75(4):1515–24. doi: 10.1002/mrm.25730 (PMC4556070; doi:10.1002/mrm.25730)
Supplement: Supplementary file 4 — Supporting Information [file MRM-75-1515-s004.docx]

Supplementary Video 1: Representative mid-ventricular single-slice time-resolved data overlaid on anatomical reference images. Shown here are the three metabolites, from left to right, pyruvate, bicarbonate and lactate in a fasted rat. The color axis for each of the three images is scaled relative to the maximum pyruvate.

Supplementary Video 2: Representative mid-ventricular single-slice time-resolved data overlaid on anatomical reference images. Shown here are the three metabolites, from left to right, pyruvate, bicarbonate and lactate in a fed rat. The color axis for each of the three images is scaled relative to the maximum pyruvate.

Supplementary Video 3: Representative mid-ventricular single-slice time-resolved data overlaid on anatomical reference images. Shown here are the three metabolites, from left to right, pyruvate, bicarbonate and lactate in a fed rat administered the PDK inhibitor DCA. The color axis for each of the three images is scaled relative to the maximum pyruvate.
